# Polydopamine encapsulated new indocyanine green theranostic nanoparticles for enhanced photothermal therapy in cervical cancer HeLa cells

**DOI:** 10.3389/fbioe.2022.984166

**Published:** 2022-09-19

**Authors:** Huimin Fan, Ting Yan, Shuang Chen, Zhong Du, Gulinigaer Alimu, Lijun Zhu, Rong Ma, Xiaohui Tang, Youqiang Heng, Nuernisha Alifu, Xueliang Zhang

**Affiliations:** ^1^ State Key Laboratory of Pathogenesis, Prevention and Treatment of High Incidence Diseases in Central Asia, School of Medical Engineering and Technology, Xinjiang Medical University, Urumqi, China; ^2^ Department of Epidemiology and Health Statistics, School of Public Health, Xinjiang Medical University, Urumqi, China; ^3^ State Key Laboratory of Pathogenesis, Prevention and Treatment of High Incidence Diseases in Central Asia, Department of Gynecology, The First Affiliated Hospital of Xinjiang Medical University, Urumqi, China; ^4^ Central Laboratory of Xinjiang Medical University, Urumqi, China; ^5^ State Key Laboratory of Pathogenesis, Prevention and Treatment of High Incidence Diseases in Central Asia, Urumqi, China

**Keywords:** cervical cancer, new indocyanine green, polydopamine, photothermal therapy, nanoparticles

## Abstract

Photothermal therapy (PTT) has attracted extensive attention in cancer treatment due to its non-invasiveness, high efficiency, and repeatability in recent years. Photothermal agents (PTAs) are the key factor for PTT. Recently, although an increasing number of PTAs have been developed, there is still a great demand for optimized photothermal nanoparticles (NPs) with low toxicity, bio-safety and stability. Herein, new indocyanine green (IR820) with near-infrared (NIR:700–1,700 nm) fluorescence emission was selected as a photothermal agent (PTA). To enhance the PTT property, IR820 was encapsulated with another kind of PTA, polydopamine (PDA) under alkaline conditions. Furthermore, to improve the biocompatibility of the NPs, methoxy polyethylene glycol amine (mPEG-NH_2_) was modified *via* a Michael addition to form a novel kind of IR820@PDA@PEG NPs. After detailed characterization and analysis, the obtained IR820@PDA@PEG NPs showed a spherical shape with an average diameter of ∼159.6 nm. Meanwhile, the formed IR820@PDA@PEG NPs exhibited better photostability and lower cytotoxicity than free IR820 molecules. The photothermal performance of IR820@PDA@PEG NPs was further analyzed *in vitro*, and the temperature of IR820@PDA@PEG NPs (100 μg/ml) reached 54.8°C under 793 nm laser irradiation. Afterwards, the cellular uptake of IR820@PDA@PEG NPs was evaluated *via* confocal laser scanning fluorescence microscopic imaging. Then, PTT experiments on HeLa cells demonstrated that IR820@PDA@PEG NPs can hyperthermal ablate cancer cells (∼49.1%) under 793 nm laser irradiation. Therefore, IR820@PDA@PEG NPs would be a promising PTA for the treatment of cervical cancer HeLa cells.

## Introduction

Although the prevention and diagnosis have made great progress (Schiffman et al., 2016; Enokida et al., 2021; Zhang et al., 2022), cervical cancer is still the second most common malignancy in females around the world (Ginsburg et al., 2017). Traditional cancer treatments such as surgery, chemotherapy and radiotherapy have certain side effects (Cohen et al., 2019; Dong et al., 2019; Lungu et al., 2019). Therefore, there is an urgent need to develop an efficient and safe treatment against cervical cancer.

PTT is a favorable strategy for tumor treatment (Nam et al., 2018; Xiao et al., 2021), which induces a local temperature rise to ablate tumors with the assistance of PTAs (Hu et al., 2020; Wang et al., 2021). PTT showed advantages of controllability, minimal invasion, and low side effects in biomedical applications (Li J. et al., 2019; Liu et al., 2020; Ren et al., 2020). It is well known that effective therapeutic effects rely on PTAs with high ability to convert light into heat energy. Until now, a variety of PTAs have been developed successively (Chen et al., 2019), including inorganic PTAs, such as semiconductors (Meng et al., 2016; Yu et al., 2017; Wang et al., 2020a; Wang et al., 2020b), metals (Nuo et al., 2018; Hong et al., 2020), metalloids (Yu et al., 2018) and carbon nanomaterials (Li J. et al., 2018); in addition, organic PTAs, such as small-molecule organic fluorescent dyes (Shirata et al., 2017; Gao et al., 2021), semiconducting polymers (Sun et al., 2019; Wang et al., 2019) and metal-organic frameworks (Yu et al., 2021; Liu et al., 2022; Yu et al., 2022), *etc*. For example, Chen et al. developed indocyanine green (ICG)-loaded and cancer cell membrane-coated nanoparticles (ICNPs) for homologous-targeting dual-modal imaging and photothermal therapy (Chen et al., 2016). Alves et al. explored a novel Hyaluronic acid functionalized nanoparticles loaded with IR780 and DOX which can be used for breast cancer chemo-photothermal therapy (Alves et al., 2019). Compared to the potential toxicity of inorganic PTAs, organic PTAs owned better biocompatibility and biodegradability. However, it should be noticed that the rapid metabolism and poor photostability of fluorescent dyes has limited their further applications in biomedicine. Therefore, it is necessary to modify organic PTAs with low biotoxicity, good stability and high photothermal conversion efficiency.

New indocyanine green (IR820) is a novel type of near-infrared fluorescence emissive organic dye (Jiao et al., 2020). As a derivative of indocyanine green (ICG) which is approved by the US. Food and Drug Administration (FDA) (Zaharie-Butucel et al., 2019), IR820 not only showed similar optical and photothermal properties as ICG, but also better stability and longer tissue retention (Hu et al., 2018; Meng et al., 2019; Chen et al., 2020). Given these advantages, IR820 has become an ideal organic PTA for PTT. For instance, Chen et al. used PEGylated melanin nanoparticles (PEG-MNPs) to synthesize a multifunctional theranostic agent, IR820-PEG-MNPs for *in vivo* detection and photothermal ablation of orthotopic micro-hepatocellular carcinoma (Chen et al., 2020). Jiang et al. developed an excipient-free nanodrug LA-IR820/DOX for synergetic chemo-photothermal therapy targeting hepatoma cells (Jiang et al., 2020). Xu et al. fabricated a p53 DNA/IR820 microneedle (MN) patch which co-loaded with p53 DNA and IR820 for synergistic gene therapy and PTT of subcutaneous tumor (Xu et al., 2020). However, there is little research on the simple and efficient preparation for the modification of IR820.

Polydopamine (PDA), as a kind of conjugated polymer, possesses great surface modification ability, good biocompatibility, photothermal ability and excellent photostability (Li Z. et al., 2019). More than that, PDA can be formed by a simple self-polymerization with adjustable size, and is a reliable candidate for the synthesis of PTT nanoprobe (Lv et al., 2017). These characteristics provide PDA a great potential to be a coating material as well as a photothermal material in biomedical applications (Li Z. et al., 2018; Zhang et al., 2019; Li et al., 2021).

In this work, IR820 was utilized and further encapsulated by PDA through dopamine polymerization under alkaline conditions. Then the PDA encapsulated IR820 was modified with mPEG-NH_2_ *via* a Michael addition. The obtained IR820@PDA@PEG NPs showed a spherical morphology with an average size of 159.6 nm. And IR820@PDA@PEG NPs possessed an absorption peak at 689 nm and fluorescence peaks at 800–1,000 nm. IR820@PDA@PEG NPs exhibited good photochemical stability in different pH solutions. In addition, CCK-8 assay and photothermal experiments demonstrated the low cytotoxicity and good photothermal properties of IR820@PDA@PEG NPs, respectively. Then, IR820@PDA@PEG NPs was further utilized for the labeling of HeLa cells. Confocal microscopic images and apoptosis assay confirmed that cellular uptake ability of IR820@PDA@PEG NPs and PTT efficiency of ∼49.1% under 793 nm laser irradiation. Therefore, our study demonstrated that IR820@PDA@PEG NPs could serve as a promising PTA candidate to realize PTT effect against cervical cancer HeLa cells.

## Experiments

### Chemicals and materials

New indocyanine green (IR820, CAS:172616-80-7) was purchased from Shanghai Sigma-Aldrich (China). Dopamine (4-(2-Aminoethyl) benzene-1,2-diol, DA, CAS:51-61-6, M.W.153.18), methoxy polyethylene glycol amine (mPEG-NH_2_, CAS: NONE6350, M.W. 2000) and Hoechst 33342 were obtained from Shanghai Macklin Biotechnology Co., Ltd (China). Tris-HCL buffer was received from Solarbio (Beijing, China). Phosphate buffered saline (PBS), high glucose dulbecco’s modified eagle medium (DMEM) and Trypsin EDTA solution (0.25%) were all supplied by Biological Industries Israel Beit Haemek Ltd. Fetal bovine serum (FBS) was received from Shanghai Hyclone Co., Ltd (China). Cell counting kit-8 (CCK-8) was provided by Beijing Biosharp Co., Ltd. FITC Annexin V apoptosis detection kit I was supplied by BD Biosciences Pharmingen Co., Ltd (United States). All chemical reagents were used as received without further purification. Deionized (DI) water purified by a Millipore system (New Jersey state, United States) was used in the experiments.

### Preparation of IR820@PDA@PEG NPs

IR820@PDA@PEG NPs was prepared by a modified method in two steps. In the first step, 10 mg of IR820 was dissolved in 10 ml of Tris buffer solution (pH = 8.5) and sonicated for 5 min. Then, 1 mg of DA was added to the reaction vial, followed by stirring (1,200 r/min) overnight at room temperature in the dark condition. IR820@PDA NPs was purified by centrifugation (16,000 r/min, 5 min) and washed thrice with DI water.

In the second step, the obtained IR820@PDA NPs was further dissolved in 2 ml of Tris solution (pH = 8.5) and then 2 mg of mPEG-NH_2_ was added. The mixture was magnetically stirred for another 24 h at room temperature in the dark condition. After that, IR820@PDA@PEG NPs was collected *via* centrifugation at 16,000 r/min for 5 min, and washed three times with DI water. The prepared IR820@PDA@PEG NPs was dispersed in DI water and stored for subsequent evaluation.

### Loading capacity (LC) and encapsulation efficiency (EE)

The LC and EE of IR820@PDA@PEG NPs were measured by a PerkinElmer UV-vis-NIR spectrometer (Lambda 750S, United States) at an absorption wavelength of 376 nm. Briefly, the absorption of free IR820 at 376 nm was measured to generate a standard curve. Then, the supernatants in two-step preparation were collected and its absorbance value was measured. Then the concentrations of IR820@PDA@PEG NPs were quantified from the standard curve. The LC and EE were calculated as follows:(Yin et al., 2020).

$$\text{LC}\;\left(\%\right)=\frac{weight\;of\;\text{IR}820\;\text{in}\;\text{NPs}}{total\;weight\;of\;\text{NPs}}\times 100\%$$

$$\text{EE}\;\left(\%\right)=\frac{weight\;of\;\text{IR}820\;\text{in}\;\text{NPs}}{weight\;of\;\text{feeding}\;\text{IR}820}\times 100\%$$

### Characterization of IR820@PDA@PEG NPs

The morphology of IR820@PDA NPs and IR820@PDA@PEG NPs were observed by transmission electron microscopy (TEM, JEM-1230, Japan). The microstructure of IR820@PDA NPs and IR820@PDA@PEG NPs were characterized by scanning electron microscopy (SEM, JSM-7610FPlus, Japan). The average size and zeta potential were measured by a Malvern Zetasizer Nano ZS-90 instrument (England). The absorption and fluorescence spectra were obtained by using a PerkinElmer UV-vis-NIR spectrometer (Lambda 750S, United States) and a Duetta fluorescence spectrometer (HORIBA, Canada), respectively. The thermal images were observed by a Fotric 323 Pro thermal camera (Shanghai Kind Electronics Co., Ltd. China).

### Stability analysis

The photochemical stability of IR820@PDA@PEG NPs was evaluated as follows: IR820@PDA@PEG NPs (10 μg/ml, 4 ml) were dispersed in PBS (pH = 5.5), PBS (pH = 6.0) and PBS (pH = 7.4), respectively. The absorption spectra were monitored on a PerkinElmer UV-vis-NIR spectrometer for 1 week.

### Photothermal performance of IR820@PDA@PEG NPs

To evaluate the photothermal performance of IR820@PDA@PEG NPs, same concentration of free IR820, PDA, mNH_2_-PEG and IR820@PDA@PEG NPs dispersions (100 μg/ml, 1 ml) were irradiated with 793 nm laser (300 mW/cm^2^) in Tris buffer, respectively. Then, the concentration-dependent temperature increase of IR820@PDA@PEG NPs were studied at different concentrations (12.5, 25, 40, 50, 80, and 100 μg/ml, 1 ml) under 793 nm laser irradiation (300 mW/cm^2^). Meanwhile, free IR820 (100 μg/ml) and IR820@PDA@PEG NPs (40, 80, and 100 μg/ml) were irradiated by 793 nm laser at different power densities (100, 200, 300, and 400 mW/cm^2^) to illustrate the dependence of temperature and power density. Moreover, after three cycles of heating and cooling, the photothermal stability was evaluated compared to free IR820.

To further assess the photothermal conversion efficiency (*η*), free IR820 (100 μg/ml, 1 ml) and IR820@PDA@PEG dispersions (100 μg/ml, 1 ml) were irradiated with a 793 nm laser at a power density of 300 mW/cm^2^, respectively. When the steady-state temperature was reached, turn laser off and cool down the solutions to room temperature. The temperature variation was monitored every 30 s with a Fotric 323 Pro thermal camera. The photothermal conversion efficiency can be calculated by equation: (Zhang et al., 2018)

$$\begin{array}{l} \eta \;\left(\%\right)=\frac{hS\left(T_{\max}-T_{surr}\right)}{I\left(1-10^{-A_{793}}\right)}\times 100\%\\ =\frac{m_{D}C_{D}\left(T_{\max}-T_{surr}\right)}{\tau_{S}I\left(1-10^{-A_{793}}\right)}\times 100\% \end{array}$$

where *h* is the heat transfer coefficient, *S* is the surface area of the container. *I* is the laser power. $m_{D}$ and $C_{D}$ are the mass and the heat capacity of solvent, respectively. $\tau_{S}$ is the time constant. *A*_*793*_ is the absorbance of the IR820@PDA@PEG NPs at 793 nm.

### Cell culture and cellular uptake

HeLa cells (a human cervical carcinoma cell line, American Type Culture Collection) were cultured in high glucose DMEM, supplemented with 10% FBS and 1% penicillin-streptomycin in a humidified incubator (37°C, 5% CO_2_).

PEG NPs only group were incubated with fresh medium and IR820@PDA@PEG NPs (80 and 100 μg/ml), respectively. HeLa cells different concentrations (40, 80 and 100 μg/ml, 600 µl) in HeLa cells was investigated by confocal laser scanning microscopy (CLSM). HeLa cells were seeded into 35 mm glass-bottom dishes (1 × 10^5^ cells per well) and incubated overnight. Then, the old medium was replaced with fresh medium containing IR820@PDA@PEG NPs. After another 2 h of incubation, HeLa cells were washed three times with 1 × PBS (pH = 7.4) and counterstained with Hoechst 33342 for 5 min in the dark condition. At last, the fluorescence images of HeLa cells were detected by a Nikon fluorescence inverted microscopy (ECLIPSE-Ti, Japan).

### *In vitro* cytotoxicity assay

The *in vitro* cytotoxicity assay of IR820@PDA@PEG NPs against HeLa cells was evaluated by the CCK-8 test. Briefly, HeLa cells were seeded in a 96-well plate (5 × 10^3^ cells per well) and incubated overnight. Then, 100 μl of fresh medium with different concentrations of free IR820 and IR820@PDA@PEG NPs (5, 10, 20 40, 60, 80, and 100 μg/ml) was added to the wells. After 4 h of incubation, CCK-8 assay was conducted according to the instructions. All experiments were performed in triplicates.

### *In vitro* PTT evaluation

To visualize the PTT effect of IR820@PDA@PEG NPs, HeLa cells were plated in 35 mm glass-bottom cell culture dishes (1 × 10^5^ cells per well) and cultured overnight. Afterwards, cell dishes were divided into four groups, including control group, laser only group, IR820@PDA@PEG NPs only group, and IR820@PDA@PEG NPs plus laser irradiation group.

HeLa cells in control group and IR820@PDA@PEG NPs only group were incubated with fresh medium and IR820@PDA@PEG NPs (80 and 100 μg/ml), respectively. HeLa cells in experiment group were treated with DMEM containing IR820@PDA@PEG NPs (80 and 100 μg/ml), and irradiated with a 793 nm laser (1 W/cm^2^, 8 min) simultaneously. Laser only group was treated with DMEM medium at the same laser condition. All cells were further incubated for 2 h. Then, cells were washed thrice and stained with Annexin V-FITC (λ_ex_ = 488 nm, λ_em_ = 800–1,000 nm) and Hoechst 33342 (λ_ex_ = 488 nm, λ_em_ = 800–1,000 nm) for 10 min. Finally, the CLSM images of the stained HeLa cells were acquired by a Nikon fluorescence inverted microscopy.

### Apoptosis assay

To further quantify the PTT effect of IR820@PDA@PEG NPs, HeLa cells were cultured and treated with DMEM only, laser only, IR820@PDA@PEG NPs only (80 and 100 μg/ml), and IR820@PDA@PEG NPs (80 and 100 μg/ml) plus laser, respectively. Then cells were incubated for 6 h. The groupings were consistent with PTT evaluation.

Apoptosis assay was conducted by Annexin V-FITC/PI double staining kit in strict accordance with the instructions. Briefly, HeLa cells were collected and washed three times with cold 1×PBS (pH = 7.4) softly. Then, the cells were resuspended in 100 µl of binding buffer, and co-incubated with 5 µl of Annexin V-FITC and 5 µl of PI for 10 min at room temperature in the dark condition. The cells were analyzed by a BD LSR II flow cytometer (BD Biosciences, United States) within 1 h.

## Results and discussion

### Preparation and characterization of IR820@PDA@PEG NPs

**SCHEME 1:**
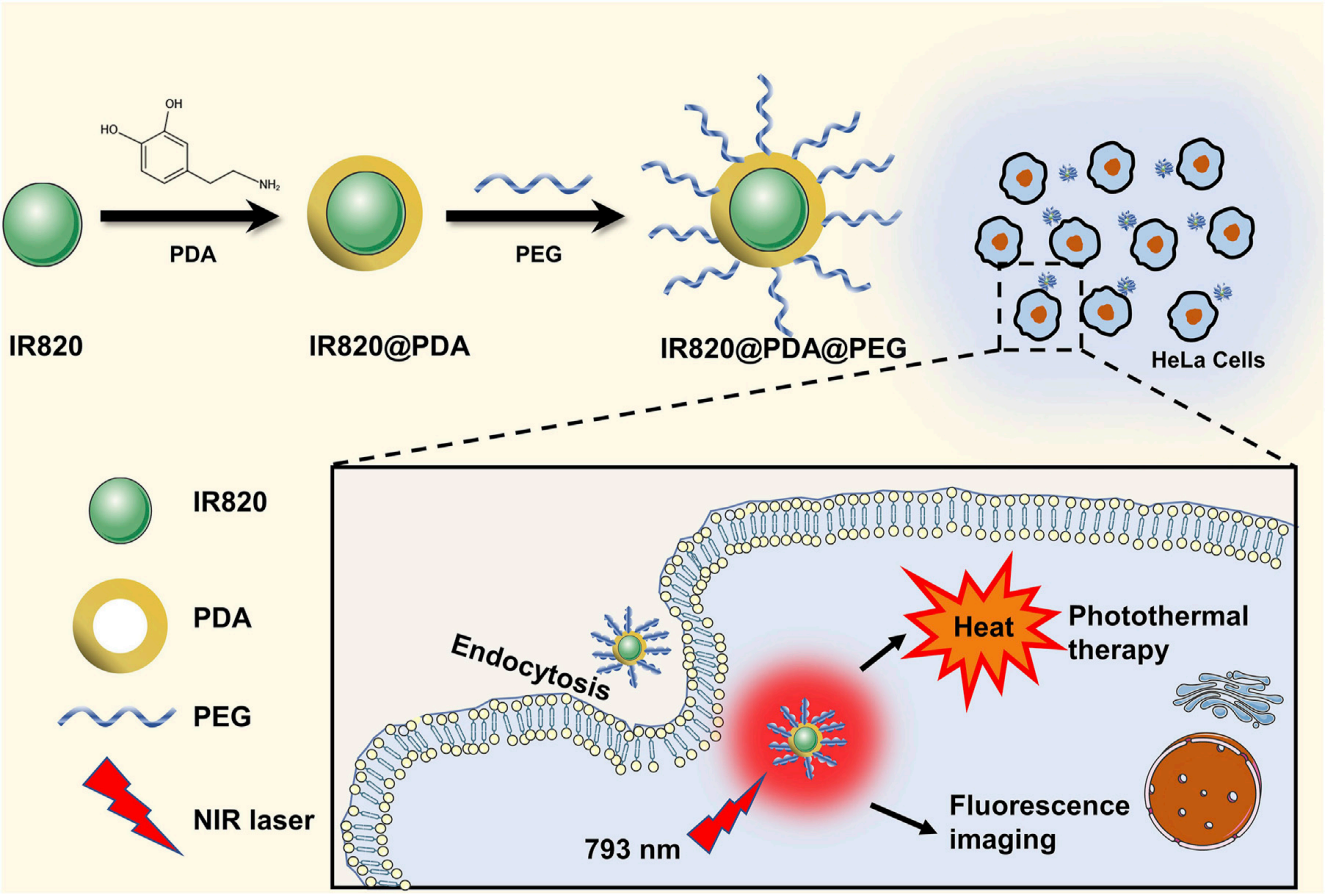
Schematic illustration of the synthesis process of IR820@PDA@PEG NPs and PTT application.

In this work, IR820@PDA@PEG NPs was fabricated *via* a two-step surface modification (Scheme 1). Firstly, PDA was utilized to encapsulate IR820 to enhance the biocompatibility and photothermal ability. IR820@PDA NPs was collected by centrifugation. Then, to further reduce the toxicity and improve the biocompatibility of IR820, mPEG-NH_2_ was modified with IR820@PDA NPs to form IR820@PDA@PEG NPs. Then the LC and EE of IR820@PDA@PEG NPs were calculated which were 62.0 and 40.0%, respectively.

**FIGURE 1:**
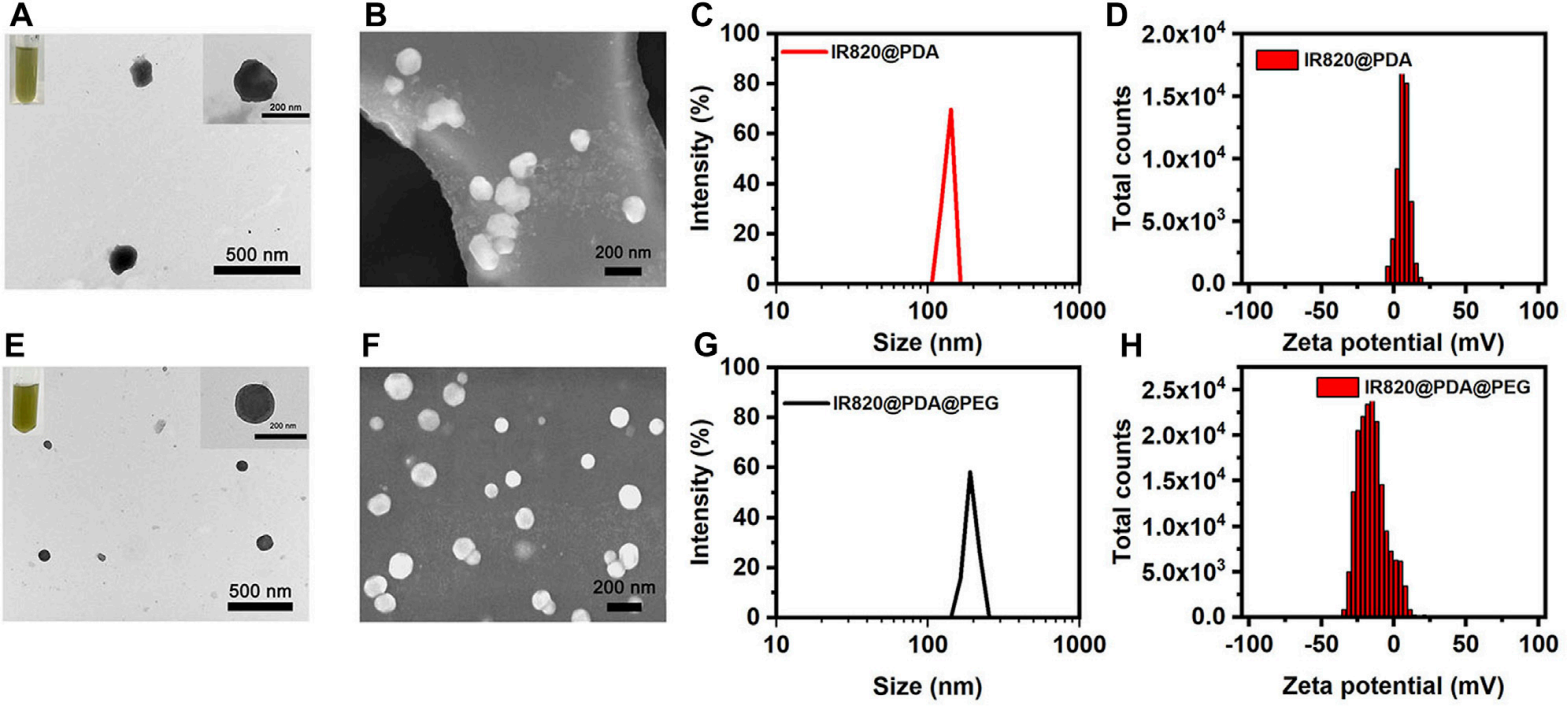
Characterization of IR820@PDA NPs and IR820@PDA@PEG NPs. **(A)** TEM (scale bar = 500 nm). **(B)** SEM (scale bar = 200 nm). **(C)** DLS result. **(D)** Zeta potential of IR820@PDA NPs. **(E)** TEM (scale bar = 500 nm). **(F)** SEM (scale bar = 200 nm). **(G)** DLS result. **(H)** Zeta potential of IR820@PDA@PEG NPs.

The shape of IR820@PDA NPs and IR820@PDA@PEG NPs were characterized by TEM and SEM. As shown in Figure 1A, the TEM images displayed that IR820@PDA NPs was spherical in shape, with size of approximately 129.5 nm in diameter. Figure 1B further illustrated the spherical structure of IR820@PDA NPs. Meanwhile, the color of the reaction solution changed to dark brown, indicating that PDA was successfully coated on IR820. And IR820@PDA NPs displayed a good dispersity in the aqueous solution after surface modification. Correspondingly, the TEM images as shown in Figure 1E demonstrated that IR820@PDA@PEG NPs exhibited a uniform spherical morphology with an average diameter of ∼159.6 nm. In addition, IR820@PDA@PEG NPs showed transparent brown color and good dispersion in aqueous solution. The SEM image further revealed that IR820@PDA@PEG NPs had a spherical shape (Figure 1F). As illustrated in Figures 1C, G, the average diameter of IR820@PDA NPs and IR820@PDA@PEG NPs measured by dynamic light scattering (DLS) were about 136.0, and 193.7 nm, respectively. Moreover, both IR820@PDA NPs and IR820@PDA@PEG NPs had a narrow size distribution. The increased size between two NPs demonstrated the successful coating of free IR820 with PDA, and the modification of IR820@PDA NPs with mPEG-NH_2_. As illustrated in Figure 1D, the zeta potential of IR820@PDA NPs was 6.54 mV. And the zeta potential of IR820@PDA@PEG NPs decreased to −15.00 mV (Figure 1H), due to the successful modification of mPEG-NH_2_. The changes of zeta potential results further confirmed that mPEG-NH_2_ was assembled with IR820@PDA NPs. As shown in Supplementary Figure S1, the zeta potential changes of each component were also measured. The zeta potentials of IR820, PDA, IR820@PDA NPs, PEG and IR820@PDA@PEG NPs were about −39.3, −48.9, −31.4, −26.0, and −15.2 mV, respectively, showing an increasing trend. These results confirmed the successful preparation of IR820@PDA NPs and IR820@PDA@PEG NPs.

### Optical characterization of IR820@PDA@PEG NPs

**FIGURE 2:**
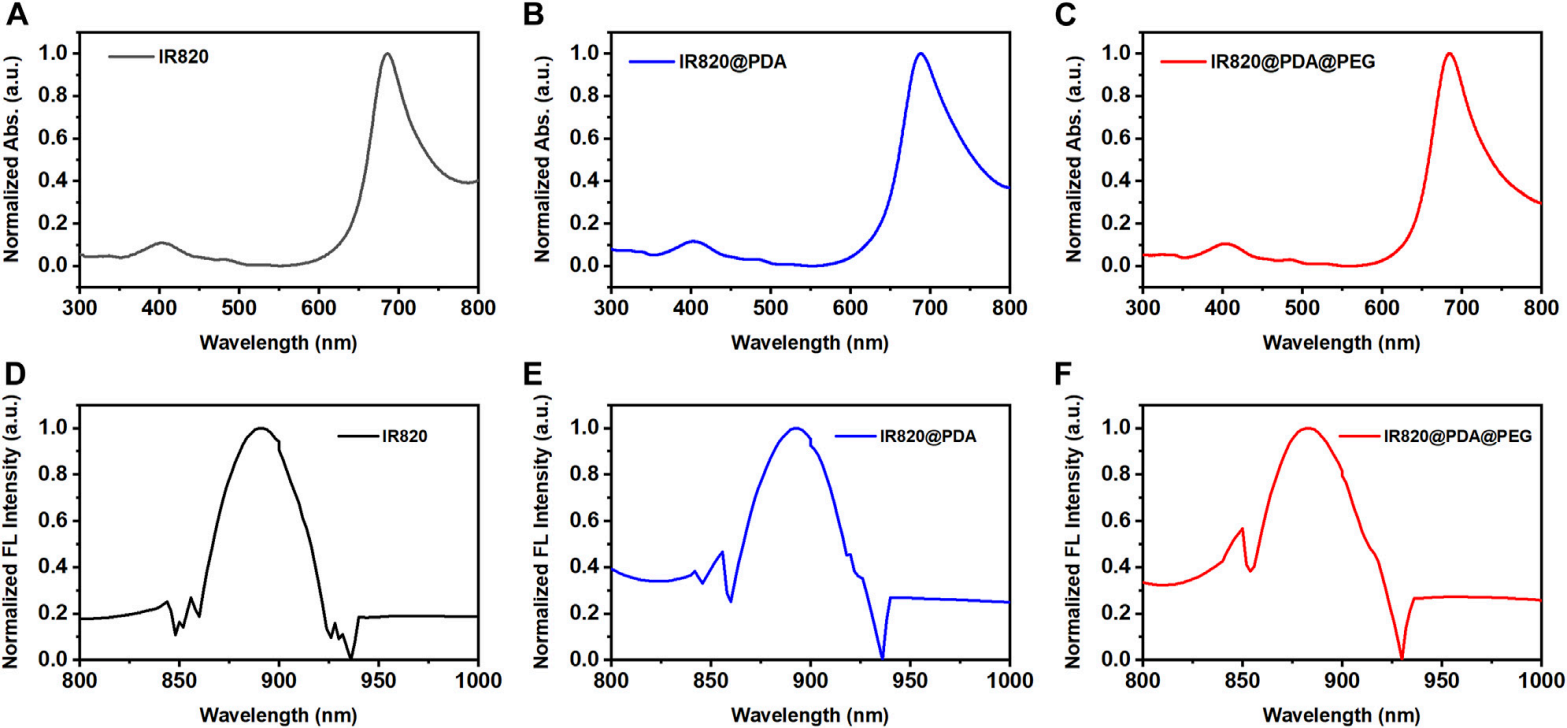
Absorption spectra of **(A)** free IR820, **(B)** IR820@PDA NPs and **(C)** IR820@PDA@PEG NPs. The normalized fluorescence spectra of **(D)** free IR820, **(E)** IR820@PDA NPs and **(F)** IR820@PDA@PEG NPs.

To improve the photothermal property, biocompatibility and stability of free IR820, PDA was utilized to encapsulate IR820, and then modified with mPEG-NH_2_. To investigate the optical characterization of IR820@PDA@PEG NPs, absorption spectra and fluorescence spectra of free IR820, IR820@PDA NPs, and IR820@PDA@PEG NPs were recorded, respectively. As shown in Figures 2A–C, the absorption peak of free IR820, IR820@PDA NPs, and IR820@PDA@PEG NPs were located at 689 nm. Besides, there was no characteristic peak in the absorption spectra of DA and PEG (Supplementary Figure S2).

The fluorescence spectra of free IR820, IR820@PDA NPs, and IR820@PDA@PEG NPs were further measured at 450 nm excitation wavelength. The spectra are normalized with respect to the fluorescence emission peaks. As shown in Figures 2D–F, the fluorescence peak of IR820, IR820@PDA NPs, and IR820@PDA@PEG NPs were measured at a range of 800–1,000 nm. The results above showed that IR820@PDA@PEG NPs have potential in bioimaging as a NIR fluorescent probe.

### Stability and CCK-8 assay

**FIGURE 3:**
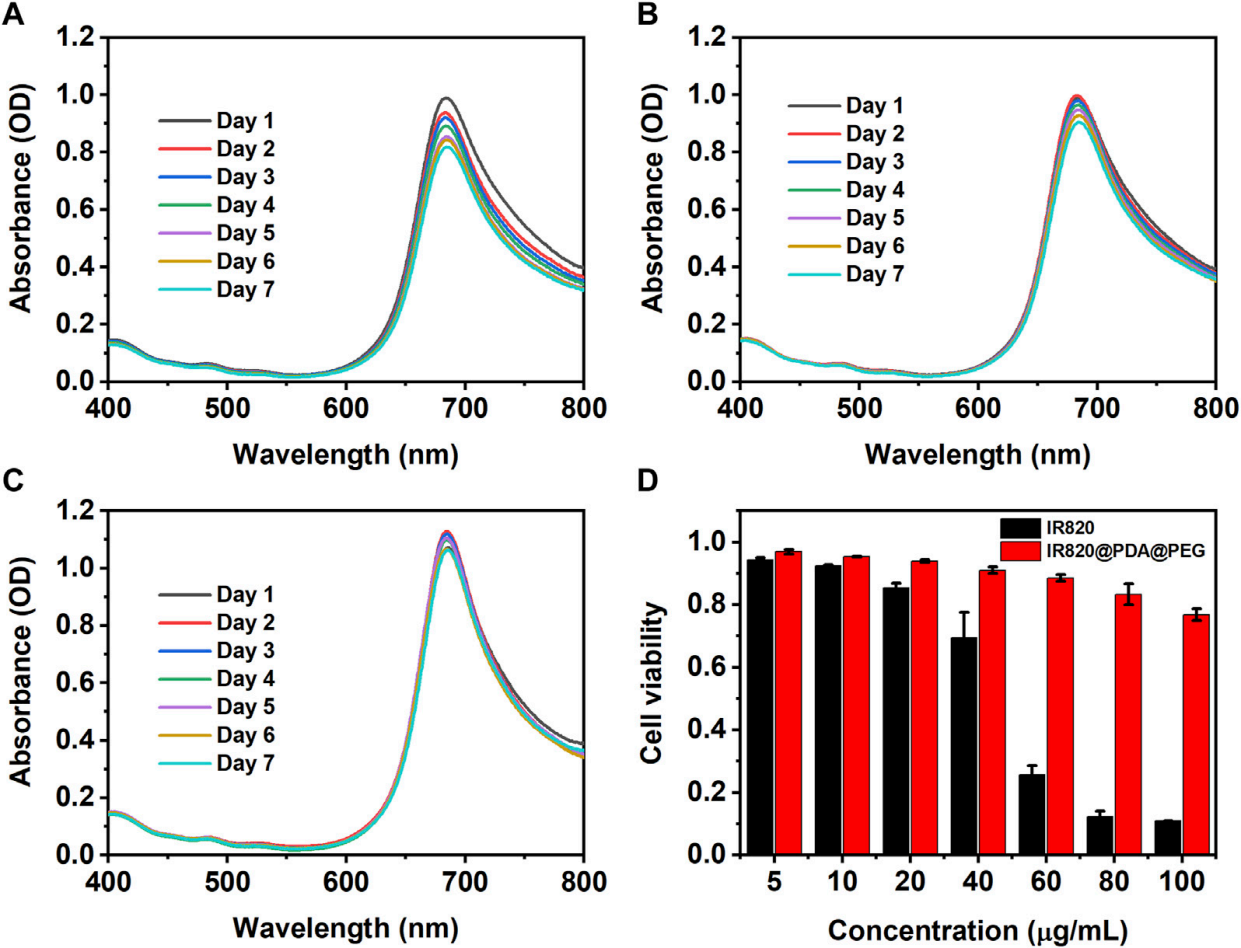
The photochemical stability of IR820@PDA@PEG NPs in pH = 5.5 **(A)**, 6.0 **(B)** and 7.4. **(C)** aqueous solutions for 7 days **(D)** Cytotoxicity of HeLa cells incubated with free IR820 and IR820@PDA@PEG NPs at different concentrations (5, 10, 20, 40, 60, 80, and 100 μg/ml) for 4 h. Data are expressed as mean S.D. (n = 3 in each concentration).

The photochemical stability of NPs is critical for their biological applications, especially for biomedical imaging and therapy. To study the photochemical stability of IR820@PDA@PEG NPs in different pH solutions (pH = 5.5, 6.0, and 7.4), the absorption peak of IR820@PDA@PEG NPs (10 μg/ml) was monitored for 7 days. As showed in Figures 3A–C, the shape of the spectra did not change in three different pH solutions. Compared to the absorption peak of the first day, the absorption peak of the other 6 days decreased slightly. Specifically, relative to the first day, the absorbance of the seventh day reduced from 0.99 to 0.82 (pH = 5.5), 0.99 to 0.90 (pH = 6.0), and 1.07 to 1.06 (pH = 7.4), respectively. The results indicated that IR820@PDA@PEG NPs retained well photochemical stability in various pH solutions.

Then the cytotoxicity of the obtained IR820@PDA@PEG NPs was evaluated by CCK-8 assay. As shown in Figure 3D, after 4 h incubation of HeLa cells with IR820@PDA@PEG NPs, the cell viabilities were obtained as 96.9%, 95.4%, 93.9%, 91.0%, 88.6%, 83.3%, and 76.8% at various concentrations of 5, 10, 20, 40, 60, 80, and 100 μg/ml, respectively. In contrast, the cell viabilities of free IR820 at the same concentrations were 92.5%, 85.4%, 69.4%, 25.7%, 12.2%, and 11.0%, respectively. The results demonstrated that IR820@PDA@PEG NPs did not cause significant cytotoxicity when the concentration was less than 100 μg/ml. Thus, IR820@PDA@PEG NPs was suitable for biomedical applications. Photothermal property evaluation of IR820@PDA@PEG NPs.

**FIGURE 4:**
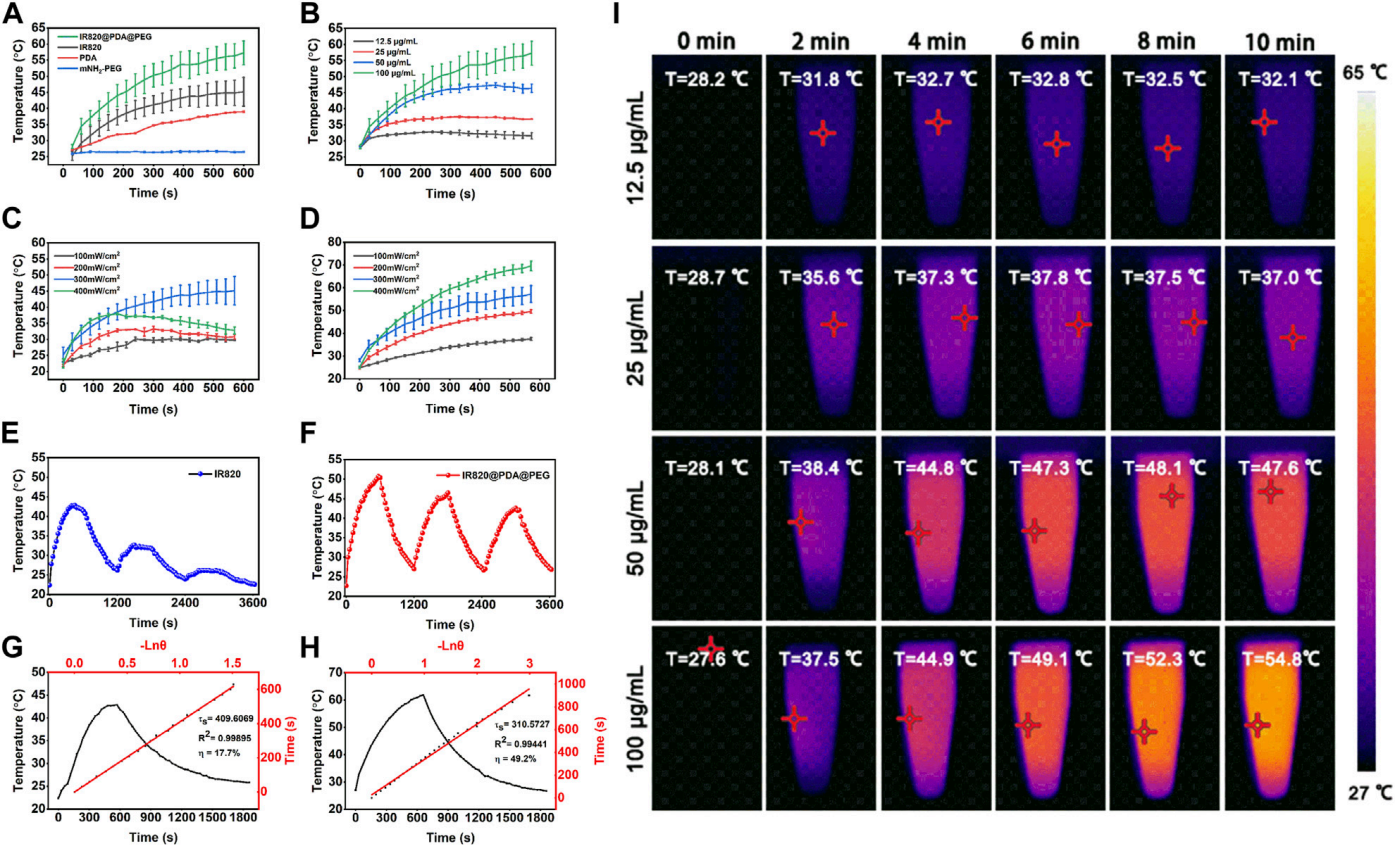
Photothermal property evaluation of IR820@PDA@PEG NPs. **(A)** Temperature curves of free IR820, mPEG-NH_2_, PDA and IR820@PDA@PEG NPs (100 μg/ml, 300 mW/cm^2^). **(B)** Temperature curves of IR820@PDA@PEG NPs at different concentrations (12.5, 25, 50, and 100 μg/ml). **(C)** Temperature curves of free IR820 under 793 nm laser irradiation with different power densities (100, 200, 300, and 400 mW/cm^2^). **(D)** Temperature curves of IR820@PDA@PEG NPs under 793 nm laser irradiation with different power densities (100, 200, 300, and 400 mW/cm^2^). **(E)** Photothermal stability of free IR820 during three heating-cooling cycles (100 μg/ml, 300 mW/cm^2^). **(F)** Photothermal stability of IR820@PDA@PEG NPs during three heating-cooling cycles 300 μg/ml, 100 mW/cm^2^). **(G)** Photothermal conversion efficiency of free IR820 (100 μg/ml, 300 mW/cm^2^). **(H)** Photothermal conversion efficiency of IR820@PDA@PEG NPs (100 μg/ml, 300 mW/cm^2^). **(I)** Thermal images of IR820@PDA@PEG NPs at various concentrations ((12.5, 25, 50, and 100 μg/ml)) under 793 nm laser irradiation at 300 mW/cm^2^ for 10 min.

To investigate the photothermal performance of IR820@PDA@PEG NPs, the temperature rise of each component was measured. As shown in Figure 4A, under the same concentration (100 μg/ml) and laser condition (793 nm, 300 mW/cm^2^, 10 min), the temperature change of IR820@PDA@PEG NPs was the fastest. Meanwhile, the concentration-dependent photothermal performance of IR820@PDA@PEG NPs was observed. As shown in Figure 4B, as concentrations increased, the temperature of IR820@PDA@PEG NPs raised from 28.0 to 31.6°C (12.5 μg/ml), from 28.7 to 36.8°C (25 μg/ml), from 28.2 to 46.4°C (50 μg/ml), and from 28.2 to 57.3°C (100 μg/ml), respectively. As shown in Figures 4C,D, excitation power density-dependent temperature changes of free IR820 and IR820@PDA@PEG NPs were monitored. At the optimized concentration (100 μg/ml) and excitation power density (300 mW/cm^2^, 10 min), the temperature rise of IR820@PDA@PEG NPs was higher than free IR820.

Then, the photothermal stability of free IR820 and IR820@PDA@PEG NPs were studied by three heat-cool cycles of laser irradiation. As illustrated in Figures 4E,F, IR820@PDA@PEG NPs exhibited better photothermal stability than free IR820. Moreover, the photothermal conversion efficiency of free IR820 and IR820@PDA@PEG NPs were observed in Figures 4G,H. The photothermal conversion efficiency (*η*) of IR820@PDA@PEG NPs was up to 49.2%, which was much higher than that of free IR820 (17.7%). Figure 4I was the photothermal images of IR820@PDA@PEG NPs at different concentrations, which illustrated the temperature and concentration-dependence of IR820@PDA@PEG NPs. As shown in Supplementary Figure S3, the temperature change of IR820@PDA@PEG NPs at different concentrations (40 and 80 μg/ml) under different power densities (100, 200, 300, and 400 mW/cm^2^) further illustrated the concentration and power dependance photothermal property of IR820@PDA@PEG NPs. These results indicated that IR820@PDA@PEG NPs owned enhanced photothermal performance and photothermal stability than free IR820. These results demonstrated that IR820@PDA@PEG NPs showed excellent photothermal performance, which laid a good foundation for further PTT studies of HeLa cells.

### Cellular uptake

**FIGURE 5:**
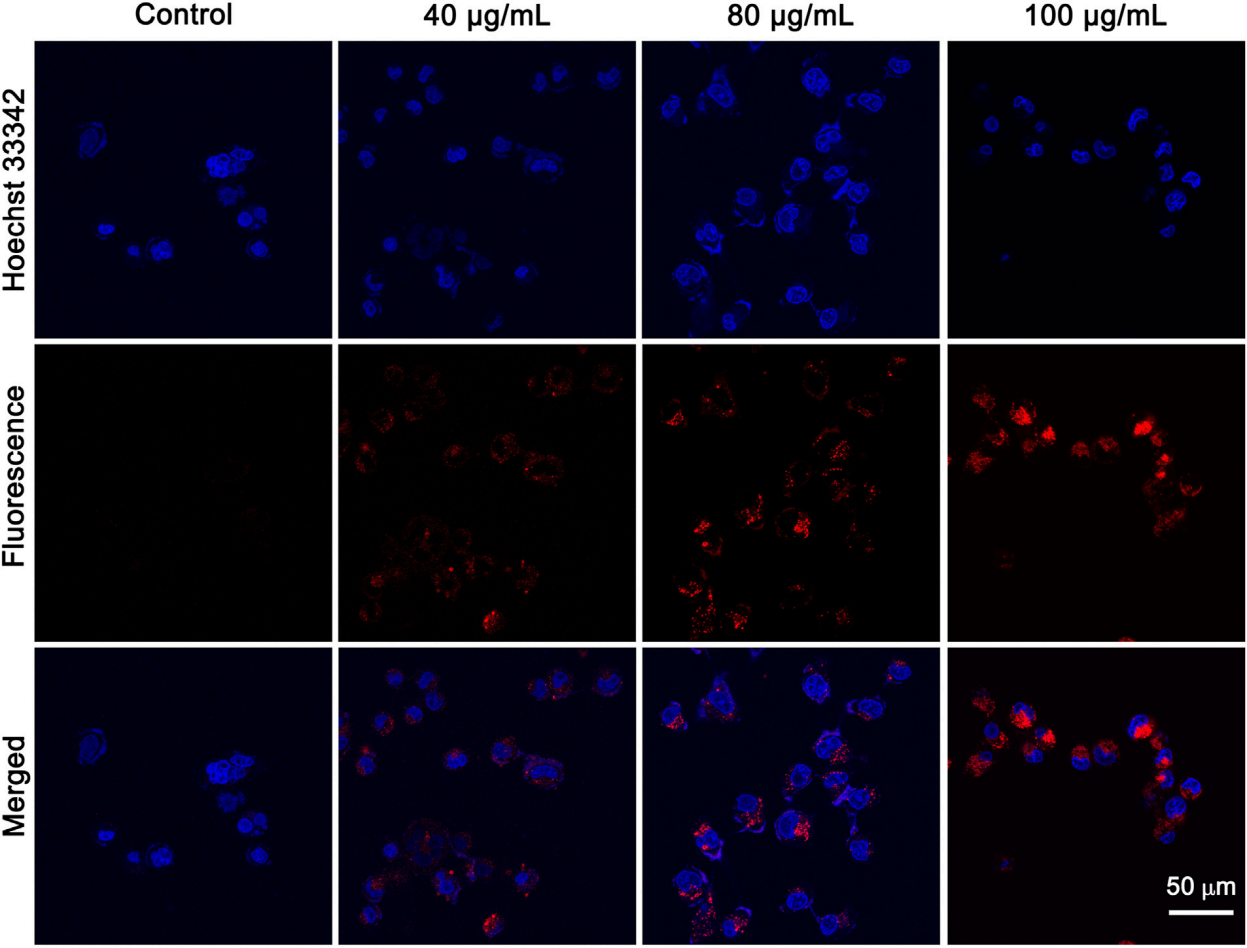
CLSM images of HeLa cells treated with DMEM, 40 and 80 μg/ml of IR820@PDA@PEG NPs. CLSM images were obtained at a ×60 objective. λ_ex_ = 640 nm, λ_em_ = 800–1,000 nm. Scale bar = 50 µm.

Effective cellular uptake of NPs is the prerequisite for efficient cancer therapy. CLSM was performed to investigate the cellular uptake ability of IR820@PDA@PEG NPs in HeLa cells. The nucleus and cytoplasm were stained with Hoechst 33342 (blue channel) and IR820@PDA@PEG NPs (red channel). HeLa cells were incubated with DMEM, 40, 80, and 100 μg/ml of IR820@PDA@PEG NPs for 2 h, respectively. As shown in Figure 5, HeLa cells treated with IR820@PDA@PEG NPs showed fluorescence signals in red channel, which demonstrated that IR820@PDA@PEG NPs can be effectively internalized by HeLa cells and was predominately presented in the cytoplasm. Besides, the fluorescence intensity in red channel with 100 μg/ml of IR820@PDA@PEG NPs group was brighter than that of 40 and 80 μg/ml, indicating that the cellular uptake of IR820@PDA@PEG NPs exhibited a concentration-dependent property. The results above illustrated that IR820@PDA@PEG NPs can be efficiently taken up by HeLa cells.

### *In vitro* PTT effect of IR820@PDA@PEG NPs

**FIGURE 6:**
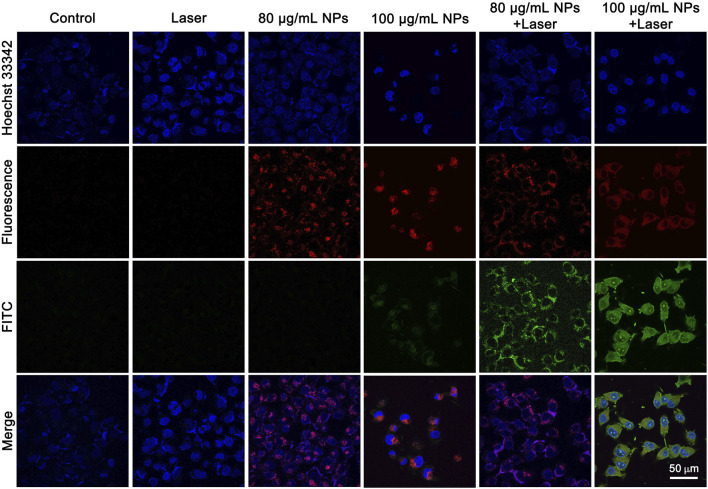
CLSM images of HeLa cells treated with DMEM only, IR820@PDA@PEG NPs only, laser only, and IR820@PDA@PEG NPs plus laser (793 nm, 1 W/cm^2^, 8 min) after stained by Hoechst 33342 and annexin V-FITC. λ_ex_ = 488 nm (Hoechst 33342). λ_ex_ = 488 nm (FITC). Images were obtained at a ×60 objective. Scale bar = 50 µm.

The PTT effect of IR820@PDA@PEG NPs on HeLa cells was analyzed by Annexin V-FITC and Hoechst 33342 staining. It is well known that cells stained with Hoechst 33342 is to visualize nucleus (blue channel) and Annexin V-FITC is to detect apoptotic cells (green channel). As shown in Figure 6, cells treated by either DMEM only or laser only exhibited fluorescence signals in blue channel, which indicated the harmlessness of 793 nm laser only (1 W/cm^2^, 8 min) to HeLa cells. And the CLSM images in IR820@PDA@PEG NPs (80 μg/ml) only group showed fluorescence signals in blue and red channels, which indicated that IR820@PDA@PEG NPs was entered into cells and owned biocompatibility. Moreover, apoptosis occurred in cells treated with IR820@PDA@PEG NPs (100 μg/ml) only. HeLa cells in IR820@PDA@PEG NPs (80 and 100 μg/ml) plus laser group showed fluorescence signals in blue, red, and green channels, verifying that IR820@PDA@PEG NPs induced apoptosis under 793 nm laser irradiation (1 W/cm^2^, 8 min). Besides, more apparently apoptotic cells were observed in 100 μg/ml of IR820@PDA@PEG NPs plus laser group compared to that of 80 μg/ml (fluorescence signals in green channel).

**FIGURE 7:**
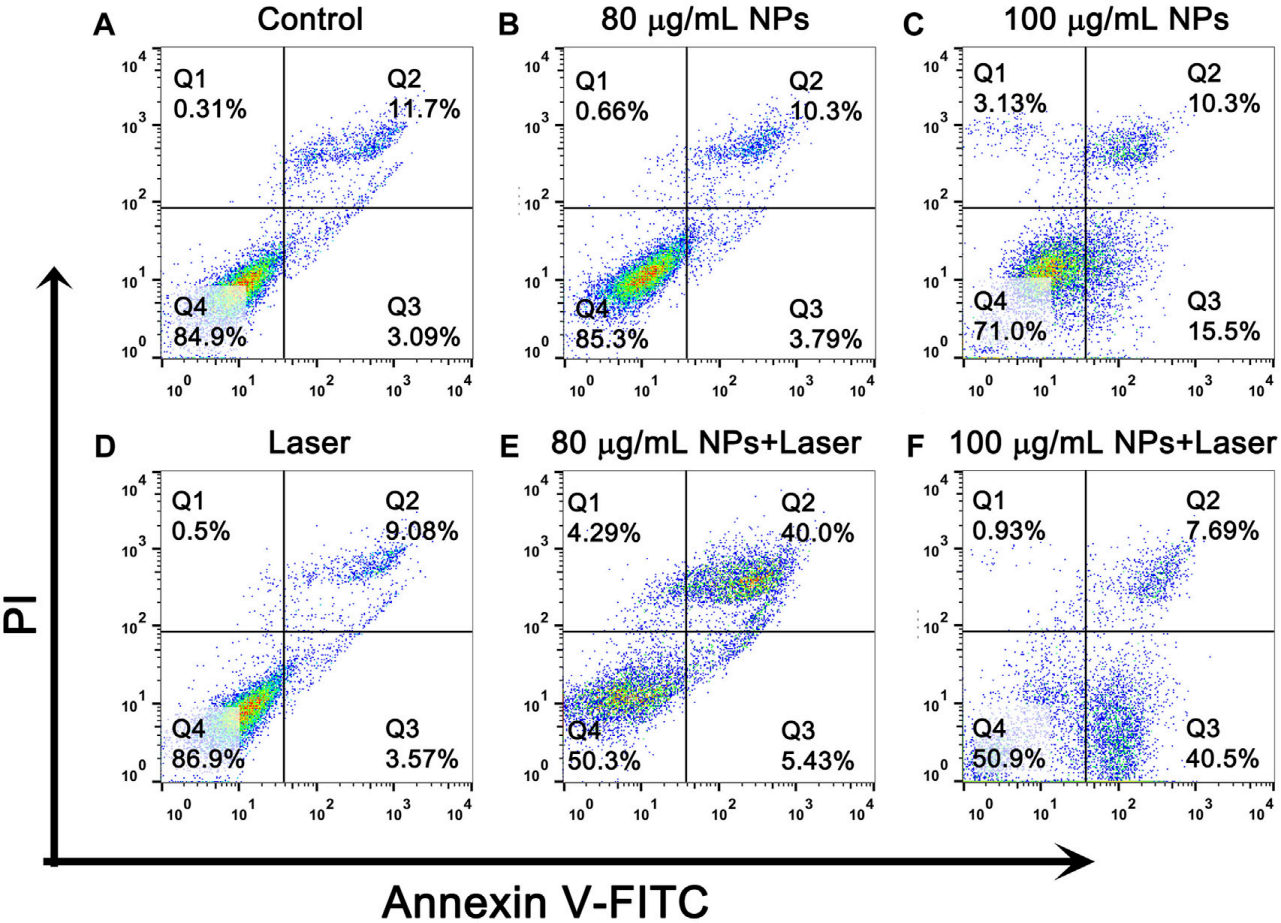
Flow cytometry analysis of HeLa cells after treatments with DMEM only **(A)**, IR820@PDA@PEG NPs only (80 and 100 μg/mL) **(B,C)**, laser only (793 nm, 1 W/cm^2^, 8 min) **(D)** and IR820@PDA@PEG NPs plus laser (793 nm, 1 W/cm^2^, 8 min) **(E,F)**.

To further quantitatively confirm the *in vitro* PTT effect of IR820@PDA@PEG NPs, cell apoptosis and necrosis were detected by apoptosis assay. As shown in Figures 7A–D, the percentage of cell death in control group, laser only group, 80 and 100 μg/ml IR820@PDA@PEG NPs group were 15.1%, 13.1%, 14.8% and 29.0%, respectively. However, the death radio in 80 and 100 μg/ml IR820@PDA@PEG NPs plus laser group was remarkably improved to 49.7% and 49.1% (Figures 7E,F). Therefore, IR820@PDA@PEG NPs had the photothermal cytotoxicity against HeLa cells under 793 nm laser irradiation. Moreover, HeLa cells incubated with IR820@PDA@PEG NPs showed massive apoptosis, demonstrating their hopeful application in the photothermal treatment of cervical cancer HeLa cells.

## Conclusion

In summary, a novel type of IR820@PDA@PEG NPs was designed and synthesized *via* a two-step surface modification. IR820@PDA@PEG NPs showed low cytotoxicity, improved biocompatibility, and super photochemical stability. In addition, IR820@PDA@PEG NPs possessed excellent photothermal performance and substantially improved photothermal conversion efficiency about 49.2% under 793 nm laser irradiation. Moreover, confocal microscopic images showed the concentration-dependent cellular labeling ability of IR820@PDA@PEG NPs. Annexin V-FITC and Hoechst 33342 staining revealed the excellent PTT of IR820@PDA@PEG NPs under 793 nm laser irradiation. The flow cytometry results further indicated the growth of HeLa cells could be effectively inhibited with the assistance of IR820@PDA@PEG NPs under 793 nm laser irradiation. Taken together, as a kind of biocompatible biomaterial, IR820@PDA@PEG NPs has a great potential for PTT against cervical cancer HeLa cells.

## Data Availability

The original contributions presented in the study are included in the article/Supplementary Material, further inquiries can be directed to the corresponding authors.
