# Supplementary material for: Polydopamine encapsulated new indocyanine green theranostic nanoparticles for enhanced photothermal therapy in cervical cancer HeLa cells
Source: Front Bioeng Biotechnol. 2022 Sep 19;10:984166. doi: 10.3389/fbioe.2022.984166 (PMC9534555; doi:10.3389/fbioe.2022.984166)
Supplement: Supplementary file 1 [file DataSheet1.PDF]

Supporting Information

Polydopamine encapsulated new indocyanine green theranostic nanoparticles for enhanced photothermal therapy in cervical cancer cells

Huimin Fan[1†], Ting Yan[2†], Shuang Chen[3], Zhong Du[3], Gulinigaer Alimu[2], Lijun Zhu[2], Rong Ma[3], Xiaohui Tang[4], Youqiang Heng[5], Nuernisha Alifu[1*], Xueliang Zhang[1*]

[1]State Key Laboratory of Pathogenesis, Prevention and Treatment of High Incidence Diseases in Central Asia, School of Medical Engineering and Technology, Xinjiang Medical University, Urumqi, China

[2]Department of Epidemiology and Health Statistics, School of Public Health, Xinjiang Medical University, Urumqi, China

[3]State Key Laboratory of Pathogenesis, Prevention, and Treatment of High Incidence Diseases in Central Asia, Department of Gynecology, The First Affiliated Hospital of Xinjiang Medical University, Urumqi, China

[4]Central laboratory of Xinjiang Medical University, Urumqi, China

[5]State Key Laboratory of Pathogenesis, Prevention and Treatment of High Incidence Diseases in Central Asia, Urumqi, China

*** Correspondence:**
Nuernisha Alifu
nens_xjmu@126.com

Xueliang Zhang
shuxue2456@126.com

[†]These authors have contributed equally to this work.

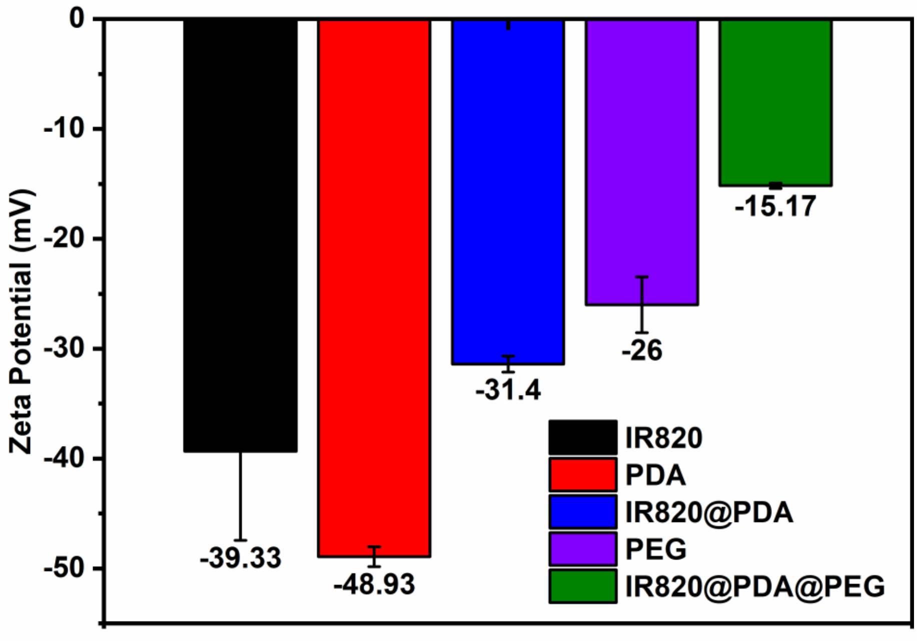

Supplementary Figure S 1 | the zeta potential changes of each component

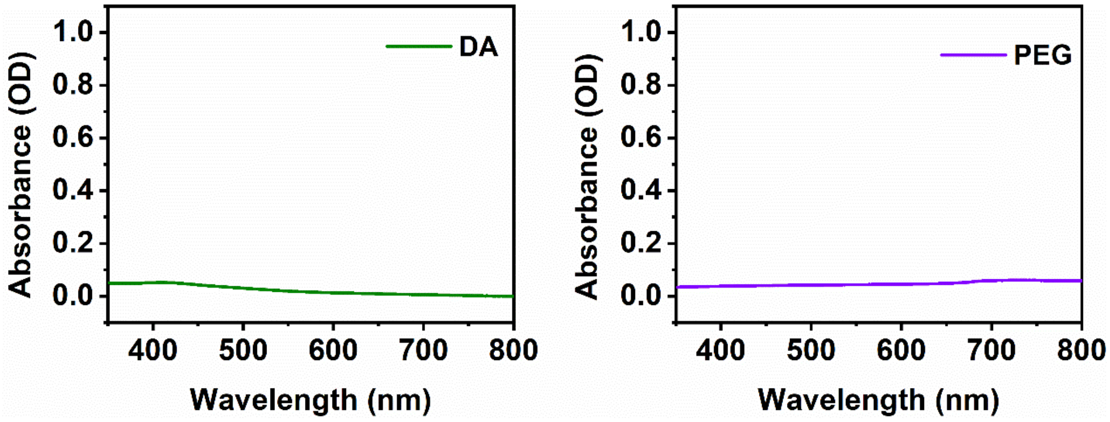

Supplementary Figure S 2 | Absorption spectra of DA and PEG.

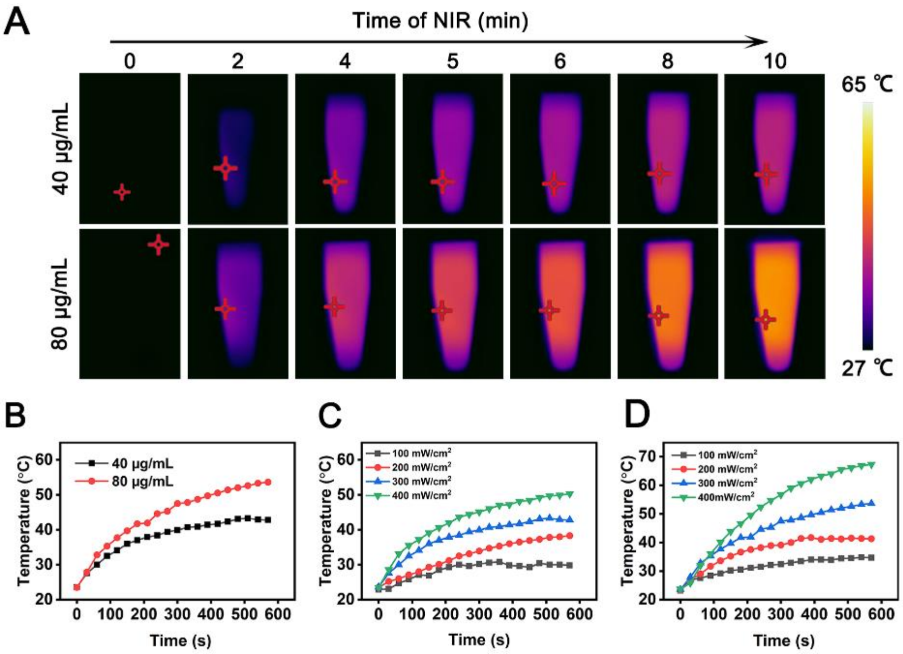

Supplementary Figure S 3 | Photothermal property evaluation of IR820@PDA@PEG NPs. (A) Thermal images of IR820@PDA@PEG NPs at different concentrations (40 and 80 μg/mL) under 793 nm laser irradiation at 300 mW/cm^2 for 10 min. (B) Temperature curves of IR820@PDA@PEG NPs at different concentrations (40 and 80 μg/mL) irradiated by 793 nm laser at 300 mW/cm^2 for 10 min. (C) Temperature curves of IR820@PDA@PEG NPs (40 μg/mL) under 793 nm laser irradiation with different power densities (100, 200, 300 and 400 mW/cm^2). (D) Temperature curves of IR820@PDA@PEG NPs (80 μg/mL) under 793 nm laser irradiation with different power densities (100, 200, 300 and 400 mW/cm^2).
